# Assessment of total retinal blood flow using Doppler Fourier Domain Optical Coherence Tomography during systemic hypercapnia and hypocapnia

**DOI:** 10.14814/phy2.12046

**Published:** 2014-07-18

**Authors:** Ayda M. Shahidi, Sunni R. Patel, David Huang, Ou Tan, John G. Flanagan, Chris Hudson

**Affiliations:** 1 Department of Ophthalmology and Vision Science, Toronto Western Research Institute, Toronto, Ontario, Canada; 2 School of Optometry and Vision Science, University of Waterloo, Waterloo, Ontario, Canada; 3 Oregon Health & Science University, Portland, Oregon

**Keywords:** Doppler Fourier Domain Optical Coherence Tomography, hypercapnia, hypocapnia, total retinal blood flow, vascular reactivity

## Abstract

The purpose of this study was to investigate changes in total retinal blood flow (RBF) using Doppler Fourier Domain Optical Coherence Tomography (Doppler FD‐OCT) in response to the manipulation of systemic partial pressure of CO_2_ (P_ET_CO_2_). Double circular Doppler blood flow scans were captured in nine healthy individuals (mean age ± standard deviation: 27.1 ± 4.1, six males) using the RTVue^™^ FD‐OCT (Optovue). P_ET_CO_2_ was manipulated using a custom‐designed computer‐controlled gas blender (RespirAct^™^) connected to a sequential gas delivery rebreathing circuit. Doppler FD‐OCT measurements were captured at baseline, during stages of hypercapnia (+5/+10/+15 mmHg P_ET_CO_2_), return to baseline and during stages of hypocapnia (−5/−10/−15 mmHg P_ET_CO_2_). Repeated measures analysis of variance (reANOVA) and Tukey's post hoc analysis were used to compare Doppler FD‐OCT measurements between the various P_ET_CO_2_ levels relative to baseline. The effect of P_ET_CO_2_ on TRBF was also investigated using linear regression models. The average RBF significantly increased by 15% (*P* < 0.0001) with an increase in P_ET_CO_2_ and decreased significantly by 10% with a decrease in P_ET_CO_2_ (*P* = 0.001). Venous velocity significantly increased by 3.11% from baseline to extreme hypercapnia (*P* < 0.001) and reduced significantly by 2.01% at extreme hypocapnia (*P* = 0.012). No significant changes were found in the average venous area measurements under hypercapnia (*P* = 0.36) or hypocapnia (*P* = 0.40). Overall, increased and decreased P_ET_CO_2_ values had a significant effect on RBF outcomes (*P* < 0.002). In healthy individuals, altered end‐tidal CO_2_ levels significantly changed RBF as measured by Doppler FD‐OCT.

## Abstract (precis)

Total retinal blood flow changes in response to the manipulation of systemic partial pressure of CO_2_ was measured in healthy individuals using Doppler Fourier Domain Optical Coherence Tomography. Increased total retinal blood flow and decreased blood flow were found in response to hypercapnia and hypocapnia, respectively.

## Introduction

Altered retinal hemodynamics and disturbance of blood flow regulation are commonly reported in ocular pathologies such as glaucoma and diabetic retinopathy; however, the overall understanding of these changes remains controversial. The need for a comprehensive understanding of the mechanisms involved in retinal blood flow regulation are required to gain further insight into the disease pathophysiology.

The development of noninvasive techniques has provided useful information on retinal hemodynamics including vessel diameter, blood velocity, and blood flow in both health and disease. Techniques such as Color Doppler imaging (CDI) can provide information on pulsatile blood flow but since the vessel diameters cannot be quantified with this technique, total retinal blood flow cannot be determined. The same limitation applies to Laser speckle techniques where blood velocity is estimated from the rate of variation of the speckle pattern. Laser Doppler velocimetry (LDV) is able to measure blood velocity in retinal arterioles and venules using the Doppler shift of light. From an additional measurement of the retinal vessel diameter, the total blood flow in a single vessel can be calculated using bidirectional LDV (Feke et al. 1989). The Canon Laser Doppler blood flowmeter (CLBF) is the only commercially available instrument that combines LDV with a retinal vessel diameter assessment system and hence provides volumetric retinal blood flow information (Riva et al. 1985; Feke et al. 1989). The limitation of the CLBF is that it measures blood flow in one vessel at a time thus does not provide total blood flow.

Optical coherence tomography (OCT) is a diagnostic imaging technology that provides high‐resolution, cross‐sectional imaging of the retinal tissue (Huang et al. 1991). Qualitative and quantitative assessments of retinal morphology via OCT are commonly used in ophthalmology for detection and monitoring of pathologies such as age‐related macular degeneration, glaucoma, and diabetic retinopathy. The new generation of OCTs, the Fourier domain (FD‐OCT), in which a high‐speed spectrometer is used to measure light echoes from all time delays simultaneously, has highly improved OCT imaging capabilities. These instruments offer a scanning speed of more than 20,000 A‐scans per second with an axial resolution of 5–7 *μ*m in the eye which has ultimately reduced the effect of motion artifacts. In addition to obtaining morphological images, FD‐OCT can also be adapted to detect the Doppler shift of reflected light, which provides information on the three‐dimensional distribution of the axial velocity component of blood in retinal vessels (Izatt et al. 1997). One of the main advantages of the technique over the existing methods of measuring retinal blood flow (e.g., the CLBF) is its ability to rapidly provide the total retinal blood flow (TRBF) by summing all measures around the optic nerve head, thereby assessing the blood flow of the whole retina rather than a single point within the retinal vascular tree. TRBF values measured by Doppler FD‐OCT in healthy people have shown good reproducibility (Konduru et al. 2012). Studies of patients with diabetic retinopathy have also shown the capability of Doppler FD‐OCT to detect differences in TRBF between patients and healthy individuals (Wang et al. 2009a).

Vascular reactivity is a quantitative measure of the ability of the blood vessels to modulate RBF in response to a provocation, such as change in systemic P_ET_O_2_ and P_ET_CO_2_. Increased systemic arterial partial pressure of O_2_ (hyperoxia) and CO_2_ (hypercapnia) results in local vasoconstriction (Gilmore et al. 2005; Tayyari et al. 2009; Sehi et al. 2012) and vasodilation (Venkataraman et al. 2008; Tayyari et al. 2009) of retinal vessels, respectively. Impaired vascular reactivity in response to changes in systemic blood gases has also been shown in patients with glaucoma (Venkataraman et al. 2010) and diabetic retinopathy (Gilmore et al. 2008; Justesen et al. 2010) prior to overt changes in blood flow. The aim of the study was to investigate changes in TRBF values measured by Doppler FD‐OCT in response to manipulation of systemic partial pressure of CO_2_ (hypercapnia and hypocapnia).

## Methods

### Participants

This study was approved by the Research Ethics Boards of University Health Network, Toronto and University of Waterloo, Canada. The study protocol adhered to the tenets of the Declaration of Helsinki. Nine healthy young individuals (mean age = 27.1 years ± 4.1**,** six males) volunteered for this study and written informed consent was obtained after the nature of the study was explained. Participants were eligible if they were nonsmoking, free of any media opacities, ocular, and systemic pathologies and were not taking any medication. Other inclusion criteria included a best‐corrected visual acuity of 0.00, or better, using the ETDRS logarithm of the minimum angle of resolution (LogMAR) acuity chart and an intraocular pressure of ≤21 mmHg. An ophthalmic examination, comprising refraction and visual acuity assessment, fundus examination using a hand held noncontact 90D Volk lens with a slit lamp and Goldman applanation tonometry, was undertaken to confirm normality. One eye for each participant was subsequently randomly selected and then dilated using a single drop of 1% tropicamide (Mydriacyl, Alcon Canada Inc, Mississauga, ON, Canada).

### Hypercapnia and hypocapnia stimuli

The partial pressures of carbon dioxide (CO_2_) and oxygen (O_2_) in end‐tidal gases were accurately varied using a custom‐designed computer‐controlled gas blender (RespirAct^™^, Thornhill, ON, Canada). The theory behind this system has been explained elsewhere (Slessarev et al. 2007) and also in a number of publications from our group (Gilmore et al. 2008; Tayyari et al. 2009). Participants breathed via a partial rebreathing circuit connected to a face mask secured to the skin using transparent medical dressing (Tegaderm; 3M, St Paul, MN) to ensure an airtight seal.

Total retinal blood flow was measured at a single visit but in two separate phases of gas provocation (i.e., hypercapnic or hypocapnic provocation) and the order of gas provocation was randomized within each phase after the establishment of each individuals baseline P_ET_O_2_ and P_ET_CO_2_ values. P_ET_CO_2_ was then modulated in 5 mmHg increments up to ±15 mmHg above or below baseline, resulting in a total of eight separate P_ET_CO_2_ conditions (i.e., baseline, ×3 hypercapnia, baseline, ×3 hypocapnia). Throughout the procedures, the end‐tidal concentration of oxygen was clamped at each individual's baseline value. The repeated baseline measurement of each individual's P_ET_O_2_ and P_ET_CO_2_ was of sufficient duration (i.e., 15–20 min) to ensure reacclimatization of the end‐tidal gases of volunteers prior to commencing the second study phase. Blood flow was repeatedly measured and pulse oxygen saturation was monitored throughout the study.

### Doppler scan acquisition and data preparation

The prototype RTVue FD‐OCT system (Optovue Inc., Fremont, CA) was employed in this study. The system takes 26,000 A‐scans per second at a depth resolution of 5 *μ*m and a transverse resolution of 15 *μ*m. The scan beam wavelength is 840 ± 10 nm, and the exposure power at the pupil is 750 *μ*W (Tan et al. 2009).

The double circular Doppler scanning pattern was employed. The scan pattern consisted of two concentric circles (inner and outer ring diameters are 3.40 and 3.75 mm, respectively), centered on the optic nerve head and transecting all retinal vessels (arterioles and venules) in the peripapillary optic nerve head, so that every vessel was sampled in two locations by the both scan rings. The Doppler angle was estimated by morphological determination of the vessel center depth difference of a given vessel between the two rings of a double circular scan. The TRBF was calculated by summing flow from all detectable branch venules. The retinal arterioles and venules have been shown to have similar blood flow based on the assumption that inflow is equal to outflow in any sealed/steady state system (Feke et al. 1989). Details of the instrument and the theory behind it have been described elsewhere (Wang et al. 2009a,b).

**Figure 1.**
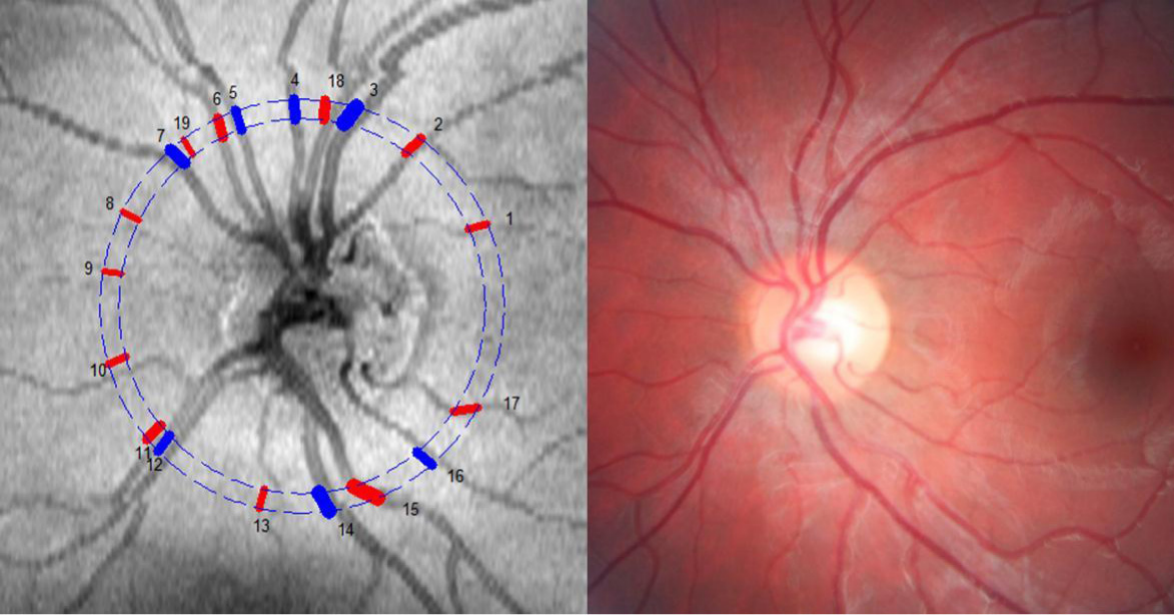
Left: Fundus image from Doppler FD‐OCT instrument for participant #9. The numbers indicate vessel location and the colors represent arterioles (red) or venules (blue). Right: Corresponding color fundus image for participant #9.

The data were imported to the Doppler OCT of Retinal Circulation (DOCTORC) software (version 3; developed by coauthors Ou Tan and David Huang) for initial automatic processing and vessel identification. Using a color fundus image for each of the participants, the scans were graded by manually refining vessel location, size, corresponding vessels in two rings, deleting extraneous vessels, and verifying vessel type (Fig. 1). Vessel diameter is measured semi‐automatedly using DOCTORC software developed by Huang and co‐workers at the Doheny Eye Institute. Briefly, vessel diameter is determined from the Doppler image in the region between the upper and lower boundary of the flow signal (Wang et al. 2011; Wang et al. 2009a). The OCT vessel diameter measurement is different from the laser Doppler flowmetry (LDF) method in a sense that in LDF, vessel diameters are measured from a fundus camera image and might appear slightly larger due to slight retinal image blur. Using DOCTORC, the vessel size is determined by the intensity shadow of the vessel on the double circular scan, one circle at a time, and then the grading on the other circle can be adjusted accordingly by manual comparison of vessel size between the circles. If the Doppler signal is clear, then sizing can be adjusted relying primarily on the size of the signal.

### Statistical analysis

The Statistical Package for Social Sciences (SPSS), version 16, was used to analyze the data. The OCT outcomes included total retinal blood flow (TRBF), venous area (VA), venous blood velocities (VV). A repeated measure analysis of variance (reANOVA) was used to compare TRFB measurements across breathing conditions. Tukey's post hoc analysis was used to determine the statistically significantly comparisons across conditions. Regression models and scatterplots were employed to investigate the effect of increasing and decreasing P_ET_CO_2_ on TRBF measurements. A *P*‐value of <0.05 was considered statistically significant except for multiple comparison analysis where a Bonferroni correction was applied and a *P*‐value of *P* < 0.008 was considered statically significant.

## Results

The TRFB data were confirmed to be normally distributed using histograms and Shapiro–Wilk (S‐W) statistics (*P* = 0.23).

### Hypercapnia

**Figure 2.**
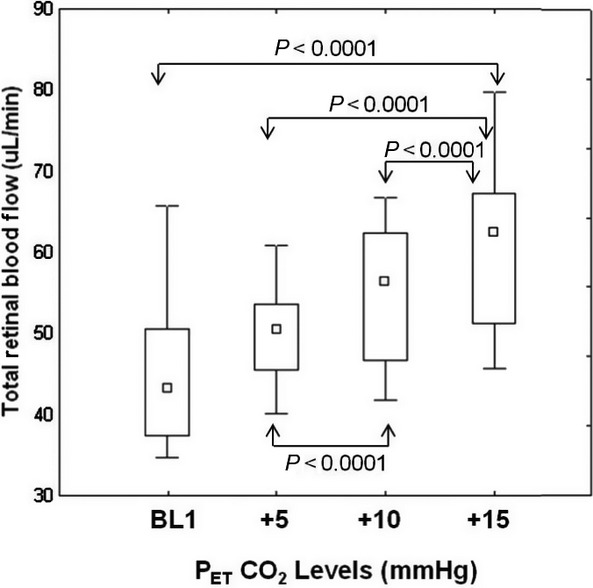
Comparisons of group total retinal blood flow relative to baseline (BL1) end‐tidal CO_2_ at three levels of increased CO_2_ (+5 to +15 mm Hg from BL1). Arrows above and below the boxes indicate significantly different pairs at *P* < 0.0001 level.

Group mean TRBF significantly increased by 15% (*F* = 65.77, *P* < 0.0001) as a result of an increase in baseline P_ET_CO_2_ to the most extreme hypercapnia (P_ET_CO_2_ +15 mmHg). Comparisons of Doppler FD‐OCT outcomes among different levels of hypercapnia showed significant increases in TRBF and VV measurements from baseline to P_ET_CO_2_ +15 mmHg. Table 1 represents the average values for Doppler FD‐OCT measurements and the detailed statistics. No statistically significant changes were found in venous area measurements with hypercapnic provocation (*P* = 0.361). Figure 2 shows alteration of TRBF in response to different levels of hypercapnia.

**Table 1.** Group mean ± standard deviations for Doppler FD‐OCT measurements at baseline and three levels of hypercapnia (+5/+10/+15 mmHg). reANOVA shows comparisons among three hypercapnia levels and baseline

Variable	Baseline 1	Hypercapnia +5 mmHg	Hypercapnia +10 mmHg	Hypercapnia +15 mmHg	reANOVA
TRBF (*μ*L/min)	45.86 ± 10.9	50.19 ± 7.19	55.11 ± 9.45	60.84 ± 12.12	***F*** **= 20.02** ***P*** **< 0.0001**
Venous					
Area (mm^2^)	0.061 ± 0.008	0.064 ± 0.01	0.065 ± 0.01	0.065 ± 0.01	*F* = 1.08 *P* = 0.361
Velocity (mm/s)	12.52 ± 1.77	13.25 ± 2.08	14.19 ± 1.04	15.63 ± 1.37	***F*** **= 17.75** ***P*** **< 0.0001**

TRBF, total retinal blood flow.

Bold values indicate statistical significance at 0.01 level.

The association between TRBF and hypercapnia revealed a significant regression effect of increasing P_ET_CO_2_ on TRBF. There was an increase of 1.22 *μ*L/min in TRBF with every 1 mmHg increase in P_ET_CO_2_ (*r*^2^ = 0.45, *F* = 27.50, *P* < 0.0001).

### Hypocapnia

**Figure 3.**
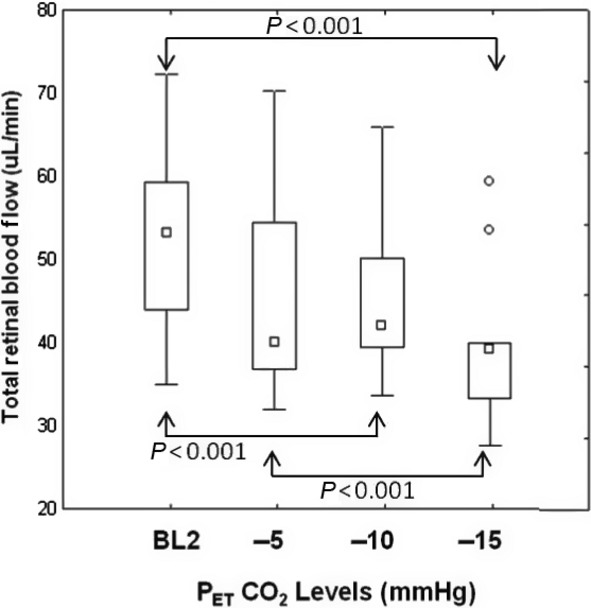
Comparisons of group total retinal blood flow relative to baseline (BL2) end‐tidal CO_2_ at three levels of decreased CO_2_ (−5 to −15 mmHg from BL2). The outliers are shown as individual round points. Arrows above and below the boxes indicate significantly different pairs at *P* < 0.001 level.

Group mean TRBF significantly decreased by 15% relative to the baseline (*F* = 8.38, *P* = 0.001) as a result of a reduction in baseline P_ET_CO_2_ to the most extreme hypocapnia (P_ET_CO_2_ −15 mmHg). Post hoc analysis, however, did show significant differences in mean TRBF at hypocapnia levels of −5, −10, and −15 mmHg (Table 2). P_ET_CO_2_ values significantly decreased from 31 mmHg at baseline to 20 mmHg at the third stage of hypocapnia (Mean ± SD: 28.22 ± 5.82 mmHg, *F* = 65.33, *P* < 0.0001; Fig. 3). Significantly reduced TRBF and VV were found with extreme hypocapnia provocation (*P* < 0.001). Moreover, venous area measurements were not found to be different among baseline and three levels of hypocapnia. Detailed statistics are shown in Table 2. As shown by the regression analyses, significantly reduced TRBF measurements were found in association with hypocapnia so that there was a 0.75 *μ*L/min decrease in TRBF with every 1 mmHg decrease in P_ET_CO_2_ (*r*^2^ = 0.12, *F* = 4.41, *P* = 0.04).

**Table 2.** Mean ± standard deviations for Doppler FD‐OCT measurements at baseline and three levels of hypocapnia (−5/−10/−15 mmHg). reANOVA shows comparisons among three hypercapnia levels and baseline

Variable	Baseline 2	Hypocapnia −5 mmHg	Hypocapnia −10 mmHg	Hypocapnia −15 mmHg	reANOVA
TRBF (*μ*L/min)	48.90 ± 14.68	45.63 ± 12.62	45.08 ± 10.35	39.75 ± 10.43	***F*** ** = 8.38** ***P*** ** = 0.001**
Venous					
Area (mm^2^)	0.061 ± 0.011	0.065 ± 0.007	0.060 ± 0.009	0.063 ± 0.018	*F* = 0.92 *P* = 0.403
Velocity (mm/s)	12.86 ± 2.80	13.70 ± 3.65	12.88 ± 2.05	10.87 ± 2.85	***F*** ** = 12.56** ***P*** ** = 0.012**

TRBF, total retinal blood flow.

Bold values indicate statistical significance at 0.01 level.

### Systemic responses to change in P_ET_CO_2_

Heart rate, systolic, and diastolic blood pressure increased significantly from baseline to extreme hypercapnia (*P* < 0.01). Otherwise, the remaining systemic parameters were unchanged throughout the whole of the protocol (Table 3). The effect of participants age and gender on total retinal blood flow at baseline P_ET_CO_2_ and P_ET_O_2_ values was assessed and no significant associations were found (*P* = 0.13 and *P* = 0.09, respectively). Mean TRBF at baseline was the same across the genders (*P* = 0.34). P_ET_O_2_ did not change throughout the whole of the protocol (Table 3).

**Table 3.** Mean ± standard deviation showing systemic responses to the gas provocation protocol

	P_ET_CO_2_	P_ET_O_2_	SPO_2_	HR	Systolic BP	Diastolic BP
Baseline 1	35 ± 2	110 ± 4	98 ± 1	76 ± 10	121 ± 10	77 ± 11
P_ET_CO_2 _+ 5	40 ± 2	110 ± 5	98 ± 1	75 ± 11	125 ± 11	79 ± 6
P_ET_CO_2 _+ 10	45 ± 2	110 ± 4	98 ± 1	79 ± 10	128 ± 11*	80 ± 7*
P_ET_CO_2 _+ 15	50 ± 2	109 ± 4	99 ± 1	82 ± 12*	135 ± 11*	81 ± 12*
Baseline 2	34 ± 2	110 ± 4	98 ± 1	76 ± 10	121 ± 11	77 ± 10
P_ET_CO_2 _− 5	30 ± 2	110 ± 4	98 ± 1	77 ± 11	121 ± 12	76 ± 6
P_ET_CO_2 _− 10	25 ± 2	110 ± 4	98 ± 1	76 ± 12	121 ± 12	77 ± 13
P_ET_CO_2 _− 15	21 ± 2	110 ± 4	98 ± 1	76 ± 11	121 ± 12	77 ± 11

P_ET_CO_2_, end‐tidal carbon dioxide; SP, systemic pulse; HR, heart rate; BP, blood pressure. Bold font & *; indicates statistical significance.

## Discussion

This study, in agreement with previous studies (Harris et al. 1995; Tayyari et al. 2009), demonstrated that hypercapnia causes an increase in RBF and has a significant positive association with RBF in healthy individuals. It was also found that venous blood velocities increased significantly with extreme hypercapnia. Conversely, it was demonstrated that hypocapnia also significantly reduced TRBF and venous velocity at the extreme levels. To our knowledge, this is the first study to investigate change in TRBF measured by Doppler FD‐OCT in response to manipulation of systemic partial pressure of CO_2_.

Retinal blood vessels are known to be sensitive to altered arterial CO_2_ pressure (Delaey and Van De Voorde 2000). Changes in end‐tidal CO_2_ are known to reflect the change in the arterial partial pressure of CO_2_ (Rhoades and Pflanzer 1992). The sequential gas breathing circuit used in this study was capable of controlled and stable, and thereby the safe administration of CO_2_ to the individuals so that an absolute ±15 mmHg change in fractional CO_2_ could be achieved while maintaining a constant fractional O_2_. This ultimately provided robust hyper‐ and hypocapnic conditions with tight control of oxygen partial pressure levels to avoid any vasoconstriction caused by altered O_2_ concentration and hence isolating the outcomes merely to P_ET_CO_2_.

The outcomes of the systemic responses to different P_ET_CO_2_ levels as presented in Table 3 showed that the group mean magnitude of blood pressure elevation associated with the most extreme hypercapnic provocations was approximately +14 to +4 mmHg for systolic and diastolic values, respectively. Hypercapnia is known to be associated with decreased vascular resistance; however, we are confident that this modest increase in blood pressure is physiological and can be adequately accommodated by autoregulatory mechanisms which inherently stabilize retinal blood flow (Venkataraman et al. 2008). Also, the significant increase in blood pressure only occurs because of the very low variability associated with our hypercapnia challenge methodology.

Previous studies that have investigated retinal vascular reactivity by means of gas provocation have shown that hypercapnia causes an increase in retinal arteriolar blood flow, velocity as well as vessel diameter (Venkataraman et al. 2006). Retinal and choroidal blood flows in animal models have shown significant reduction during hypocapnia (PaCO_2_ 20–25 mmHg) (Wang et al. 2008). The effect of hypocapnia on human ocular circulation and visual function levels has also been studied mainly in association with exhaustive exercise and hyperventilation and has showed a decrease in ocular blood flow (Ikemura and Hayashi 2012).

The range of total retinal blood flow values found in this study was 35.8–65.7 (*μ*L/min) and this was within the range of previously reported values. Using the same technique, Wang et al. (2009a,b) reported a range of 40.8–60.2 (*μ*L/min) for total venous blood flow, whereas Hwang et al. (2012) reported an average TRBF of 45.5 ± 9.5 *μ*L/min. Other published average venous blood flow values measured by the bidirectional laser Doppler velocimetry (BLDV) include 34.0 ± 6.3 *μ*L/min (Riva et al. 1985) and 64.9 ± 12.8 *μ*L/min using the CLBF (Riva et al. 1985; Garcia et al. 2002) that are also generally in agreement with our findings. It is noteworthy that previous studies utilizing hypercapnic provocation to assess ocular vascular reactivity have not reported taking any measures to maintain FeO_2_ at a constant value. As previously mentioned, in this study, the use of the sequential rebreathing circuit resulted in a physiologically insignificant change in P_ET_O_2_, that is, a near isoxic hypercapnic provocation.

The current Doppler FD‐OCT system measures the TRBF based on venous flow since arteriolar and venous flow must be equivalent in a sealed fluid system. The magnitude and exaggerated pulsatility of the arteriolar blood flow result in relatively high measurement variability (Wang et al. 2009a,b). The magnitude of arteriolar velocity can also exceed the reliable detector range of the instrument (Bonner and Nossal 1990). The current results showed that the magnitude of increase in retinal venular diameter, blood velocity, and blood flow in response to a 15% increase in hypercapnia relative to baseline (i.e., a typical increase of P_ET_CO_2_ from 38 to 44 mmHg) was +6.3%, +24% and +32%, respectively. These outcomes are comparable and consistent with previously published work from our laboratory on retinal arteriolar changes that utilized a 15% increase of hypercapnia relative to baseline (i.e., +3.2%, +26.4%, and +34.9% for diameter, velocity, and flow, respectively) (Venkataraman et al. 2006) that ultimately confirm the equivalency of the venular and arteriolar flows. It is also noteworthy that based on these reported retinal blood flow values under hypercapnia*,* the average blood flow in healthy participants significantly increased from 7.9 *μ*L/min at baseline to 10.7 *μ*L/min at a 15% increase in hypercapnia relative to baseline with a standard deviation of approximately 2 *μ*L/min. Therefore, a minimum sample size of 6 was required per group (or in this case breathing stage) to reach a power of 0.90 to find this significant difference in our study, with an alpha value of 0.05. Our study sample as mentioned in our methods stands at 9. Moreover, post‐power analysis showed that with an alpha value of 0.05 for the current sample size of 9, the power is calculated to be 0.98 that is very strong.

With respect to changes in other vascular parameters, the previously reported (Wang et al. 2009a,b) range for venous area was between 0.033 and 0.065 mm^2^ which accommodates the average values found at baseline in this study (0.062 and 0.061 mm^2^ at baseline 1 and 2, respectively). The baseline venous velocity values in this study ranged from 10.06 to 15.66 (average 12.70 ± 1.51 mm/s) which compared to previously FD‐OCT reported values which are slightly lower (17.7 ± 3.1 mm/s) (Wang et al. 2011). An advantage of Doppler FD‐OCT over the LDV technique is that it provides velocity information over a whole vessel cross section with the phase tomogram and hence is less influenced by fixation errors (Werkmeister et al. 2012). Overall, these results indicate the compatibility of the outcomes produced by the Doppler FD‐OCT system with those from already established methods for measuring retinal blood flow and vascular reactivity. It is noteworthy that, unlike the velocity outcomes, the vessel area results did not reveal any changes under various CO_2_ levels. Despite reports on good reproducibility of Doppler FD‐OCT‐derived TRBF, the venous area measurements did not significantly change during either hypercapnic or hypocapnic provocation, while TRBF and VV did change as anticipated (Wang et al. 2011; Konduru et al. 2012). This can be explained by the multiple sites and subjective determination of venular area using Doppler FD‐OCT methodology. The current Doppler FD‐OCT instrument calculates TRBF of the major retinal venules coursing toward the ONH and these venules are known to have higher magnitudes of vascular reactivity than the retinal arterioles.

Early diagnosis of ocular diseases that are associated with blood flow regulation abnormalities is essential for effective treatments. Doppler FD‐OCT has proved to be capable of detecting blood flow changes provoked by breathing different gases at various concentrations. There are also still some limitations to the double circular scanning method that need to be addressed in the future development of the technique. These include complete elimination of eye motion which can resolve possible errors in Doppler angle measurement, and full automation of the software for objective and reliable delineation and detection of vessel area. Larger studies are required to assess the capability of the current Doppler FD‐OCT system to detect differences in vascular reactivity in an older cohort as well as in disease groups. Recently, other techniques have been developed to measure retinal blood flow using Doppler OCT. One system uses a special bidirectional system OCT with two simultaneous beams with a fixed angle offset. The difference in the Doppler shift between the two beams and the angle between them is then used to calculate absolute blood velocity (Dai et al. 2013; Doblhoff‐Dier et al. 2014). Another system uses raster scanning and swept source OCT (Baumann et al. 2011; Choi et al. 2012). The flow is calculated by integrating Doppler shift over the vessel area in an en face plane. None of these techniques require measurement of Doppler angle in postprocessing, thus improve the reliability of TRBF by excluding noise from estimation of Doppler angle. Moreover, these techniques require special features including dual beam OCT or faster scanning OCT which facilitates capturing multiple volume scans in 1 sec. These features, however, do not exist in current clinical OCT systems in the market. Despite the limitations, the flow profile in Doppler FD‐OCT can be characterized across both depth and transverse dimensions and measurements of TRBF do not require any assumptions regarding vessel shape or flow profile characteristics (Wang et al. 2011).

Doppler FD‐OCT can be potentially used as an objective tool to measure retinal blood flow in ocular diseases in clinical settings and to enhance the understanding of pathophysiology of retinal vascular dysregulation.

## Acknowledgments

Ontario Research Fund (ORF).

## Conflict of Interest

Oregon Health & Science University (OHSU), Dr. Huang, and Dr. Tan have a significant financial interest in Optovue, a company that may have a commercial interest in the results of this research and technology.
